# Use of confocal laser endomicroscopy to predict relapse of ulcerative colitis

**DOI:** 10.1186/1471-230X-14-45

**Published:** 2014-03-11

**Authors:** Chang-Qing Li, Jun Liu, Rui Ji, Zhen Li, Xiang-Jun Xie, Yan-Qing Li

**Affiliations:** 1 Department of Gastroenterology, Laboratory of Translational Gastroenterology, Shandong University Qilu Hospital, No. 107, Wenhuaxi Road, Jinan, Shandong 250012, China; 2 Department of Gastroenterology, Qingdao Municipal Hospital, Qingdao, China

**Keywords:** Ulcerative colitis, Relapse, Confocal laser endomicroscopy, UC, CLE

## Abstract

**Background:**

Assessment of inflammatory activity in patients with ulcerative colitis (UC) is crucial to the prediction of relapse. Confocal laser endomicroscopy (CLE) is an accurate tool for assessing inflammatory activity in UC patients. This study aimed to evaluate whether CLE could be used to predict UC relapse reliably.

**Methods:**

In total, forty-three patients with documented UC were analyzed in this study. Patients identified as having obvious active inflammation by conventional colonoscopy were excluded. The mucosa of each patient’s sigmoid colon and rectum was assessed by CLE before targeted biopsies were taken. The patients were then followed up for at least 12 months to evaluate relapse according to the Simple Clinical Colitis Activity Index. The correlation between CLE classification and UC relapse was evaluated.

**Results:**

Seventeen of 20 patients with histologically confirmed normal or chronic inflammation were diagnosed as having non-active inflammation by real-time CLE and 22 of 23 patients with histologically confirmed acute inflammation were diagnosed as having active inflammation by CLE. The sensitivity, specificity, and accuracy of CLE in real-time diagnosis of active inflammation were 95.7%, 85%, and 90.7%, respectively. The agreement between CLE and conventional histology was excellent (kappa value = 0.812). Two of 18 (11.1%) patients who were classified as having non-active inflammation by CLE relapsed, while 16 of 25 (64%) patients classified as having as active inflammation relapsed. The relapse rate of patients with active inflammation was significantly higher than of those with non-active inflammation (P < 0.001).

**Conclusions:**

CLE is comparable to conventional histology in predicting relapse in patients with UC.

## Background

Disease duration of ulcerative colitis (UC) is an independent risk factor for colorectal cancer (CRC) [1]. Since evidence shows that 5-aminosalicylate (5-ASA) can be used to prevent patients from developing CRC [2], prediction of relapse is crucial for patients who need prolonged and strengthened pharmaceutical therapy. Histological evidence currently suggests that active inflammation and basal plasmacytosis in colonic biopsies are the most reliable parameters for prediction of relapse [3]. A recent study by Bessissow et al. found that the presence of basal plasmacytosis and a Geboes Index (GI) score of ≥3.1 predicted relapse of UC with normal endoscopy [4].

With this background, the term “mucosal healing” was introduced as the goal of UC treatment. Mucosal healing is thought to be associated with an altered natural history of UC, including sustained clinical remission, reduced hospitalization, and surgery [5-7]. As clinical symptoms and signs are not well correlated with histological mucosal healing, endoscopic assessment is crucial in the management of UC, secondary to gold standard histological assessment.

There is currently no validated definition of mucosal healing with regard to colonoscopy. Limited by its lack of definition, conventional colonoscopy is sometimes unreliable for assessing mucosal healing. Although multiple scoring systems have been applied to the endoscopic assessment of mucosal healing, the applications have been limited by high inter-observer variability [8]. Reports suggest that advanced endoscopy, such as magnifying chromoendoscopy, is superior to conventional endoscopy in evaluating the inflammatory activity of UC [9], and magnifying colonoscopy is useful for predicting relapse of patients with quiescent UC [10]. It can be concluded that assessment would be more accurate if advanced colonoscopy with higher definition and magnification was used. In recent years, the optical biopsy instrument confocal laser endomicroscopy (CLE) has been introduced and validated for the practice of gastrointestinal endoscopy. With the capability of a histological level of definition and magnification, it has proved to be promising in the real-time assessment of inflammatory activity in UC [11-14].

The aim of this study was to prospectively evaluate whether the real-time active inflammation assessed by CLE is related to the higher relapse rate in patients with UC.

## Methods

### Inclusion and exclusion criteria

Patients with documented UC under colonoscopic surveillance from January 1 to June 31, 2011 were recruited into this study. Those fulfilling the criteria for clinical remission of UC according to the Simple Clinical Colitis Activity Index (SCCAI) were included [15]. The exclusion criteria were: patients younger than 18 years or older than 80 years; finding of active inflammation during colonoscopy, such as erosion, ulcer, or spontaneous bleeding; poor bowel preparation; cecum intubation failure due to bowel stricture; unwillingness to participate in this study; and contraindications to CLE, such as fluorescein allergy, hepatic or renal dysfunction, jaundice, pregnancy and/or breast feeding.

### Endoscopic procedures

Bowel preparation before CLE did not differ from that used for conventional colonoscopy. The CLE device used was an EC3870K (Pentax, Tokyo, Japan). All patients were given intravenous injections of 1 ml of 2% fluorescein sodium (Baiyunshan Mingxing Pharmaceutical, Guangzhou, China) as an allergy test before procedures were carried out. After successful cecal intubation, 5 ml of 10% fluorescein sodium was intravenously injected. The CLE procedure did not differ from that of conventional colonoscopy, except for the additional evaluation of mucosal inflammation in the distal colon, including the sigmoid colon and rectum by CLE distal laser probe.

**Figure 1:**
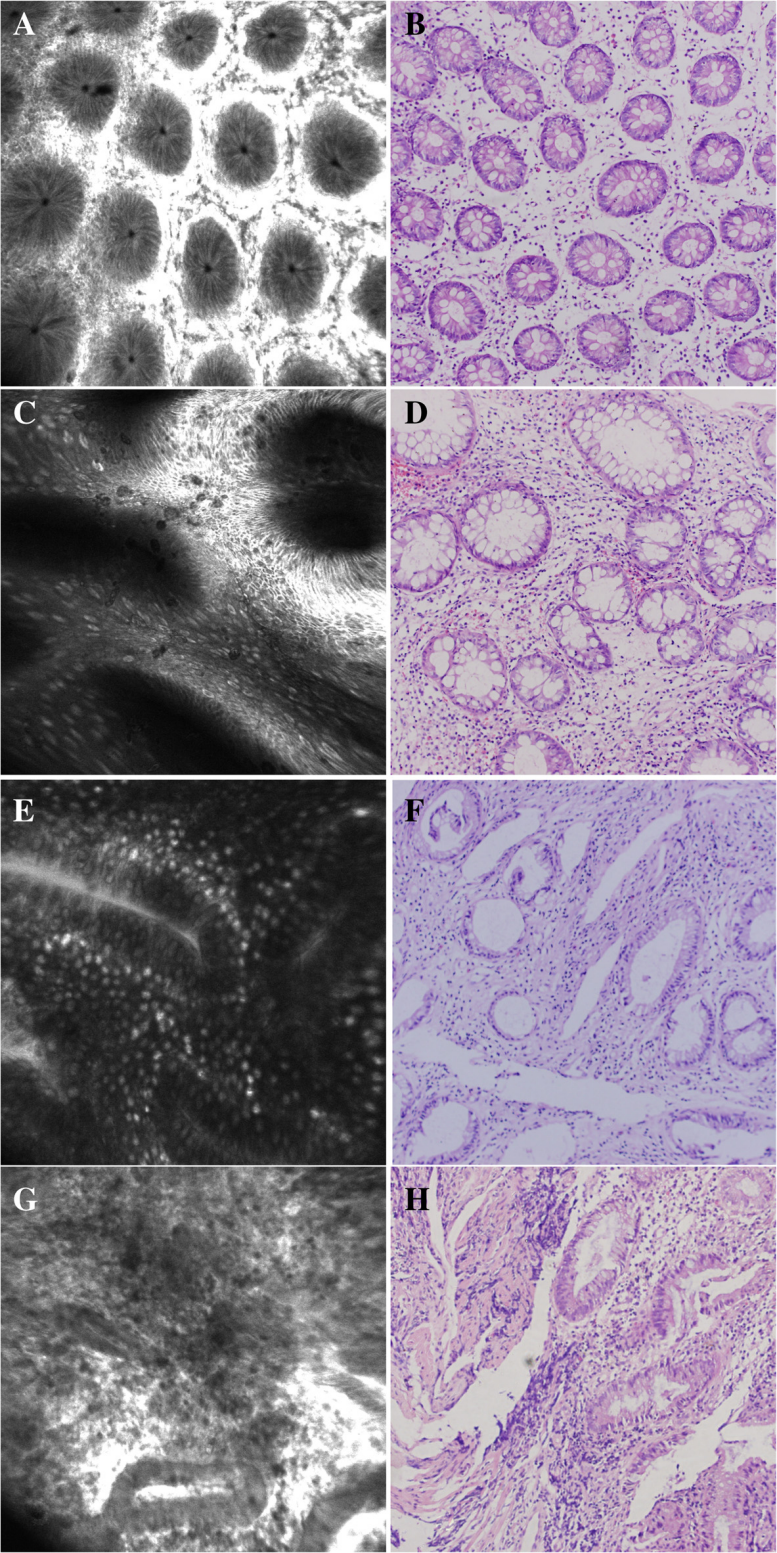
**Confocal laser endomicroscopy (CLE) crypt architecture (A, C, E and G) and conventional histology (B, D, F and H). A** and **B**: CLE image and histology of normal colonic mucosa show regular-shaped and distributed crypts. **C** and **D**: CLE image and histology of chronic inflammation of colonic mucosa show irregular-shaped, distributed but intact crypts. **E** and **F**: CLE image and histology show more dilated crypts with fluorescein leakage into the lumen, with the epithelium disrupted in some crypts. **G** and **H**: CLE image and histology show most of the crypts are disrupted.

Assessment of inflammatory activity by CLE was based on the previously published four-grade classification, in which colonic crypts were classified into four grades A, B, C and D. Types A and B are considered as normal and chronic inflammation, respectively, and types C and D indicate acute inflammation. Details of the crypt architecture classification system are illustrated in Figure 1. In addition to crypt architecture, fluorescein leakage into the lumen is also recommended as a marker of active inflammation. In CLE images of normal colonic mucosa, the lumen of the crypt is free of fluorescein and appears as a dark center in the crypt; however, in inflamed mucosa, fluorescein leaks into the crypt lumen; therefore, the lumen is brighter than the surrounding epithelium [13].

### Histology

Biopsies were taken from the areas of mucosa investigated by CLE. Biopsy specimens were fixed with 10% formalin and embedded in paraffin, and sections were stained with hematoxylin and eosin for histopathological examination. Inflammatory activity was assessed according to the GI. The GI includes six grades: structural (architectural changes): chronic inflammatory infiltrates: lamina propria neutrophils and eosinophils: neutrophils in the epithelium: crypt destruction; and erosion or ulceration. Each grade is divided into 4 or 5 subgroups. The final grades are then divided into two groups: grades ≤3.0 and >3.0, as grade 3.1 indicates neutrophils in the epithelium, a hallmark of acute inflammation [16].

### Follow-up

All the patients were followed up for 12 months. Each patient was given copies of a questionnaire containing SSCAI content in their local language (Chinese). Patients were required to return the questionnaire each week after the CLE procedures. According to the published criteria, a score of 5 or more indicates a UC relapse [17]. Details of the SSCAI are showed in Table 1.

**Table 1:** The Simple Clinical Colitis Activity Index (SCCAI)

**Symptom**	**Score**
Bowel frequency (per day)	
1-3	0
4-6	1
7-9	2
>9	3
Bowel frequency (per night)	
0	0
1-3	1
4-6	2
Urgency of defecation	
None	0
Hurry	1
Immediately	2
Incontinence	3
Blood in stool	
None	0
Trace	1
Occasionally frank	2
Usually frank	3
General well-being	
Very well	0
Slightly below par	1
Poor	2
Very poor	3
Terrible	4
Extracolonic manifestations	
(Uveitis, pyoderma gangrenosum, erythema nodusum, arthropathy)	1 per manifestation

### Statistics

Continuous variables, such as age and disease duration are presented as mean ± SD. GI score was also calculated as a continuous variable and presented as the mean (95% confidence interval [CI]). A one-way analysis of variance was used to calculate differences between continuous variables among patients. A chi-square test was used to evaluate the difference of relapse rate between groups. Relapse states were evaluated by the Kaplan–Meier survival curve. A P-value of <0.05 was taken as significant difference. All the statistical processes were run by the SPSS 16.0 program (SPSS Inc., Chicago, USA).

### Ethical considerations

The colonoscopy and biopsy procedures used in this study are standard procedures for patients with UC. The intravenously injected contrast agent fluorescein sodium is safe and approved for clinical use. All participants were informed about the purpose of this study and asked to give their informed consent. This study was approved by the Clinical Ethics Committee of Shandong University Qilu Hospital.

## Results

### Patients

Seventy-three patients with a documented history of UC were recruited into this study. Eleven patients with active inflammation at baseline were excluded (SCCAI score of 5 or more). Seventeen patients were excluded during the CLE procedures for active inflammation under white-light mode (erosion, spontaneous bleeding, and/or clear ulceration). Two patients failed to return the questionnaire during the follow-up period. In the end, 43 patients were eligible for analysis (29 males and 14 females; average age 44 years, range 19-78 years). The average disease duration was 32.5 months, range 6-72 months. The cecum of all patients was successfully intubated during the procedures. As maintenance therapy, 39 patients received 5-ASA (Mesalazine, Ethypharm Industries, France) 2-3 g daily, and 4 patients received sulfasalazine (SASP, Jialin Pharmacy, Beijing, China) 2 g daily. Patient data are given in Table 2.

**Table 2:** Demographic and clinical data of patients

**Numbers**	
Recruited	73
Excluded	30 (11 for SSCAI score of ≥5, 17 active inflammation under white-light endoscopy, 2 failed to return questionnaire)
Included	43
Gender	29 males, 14 females
Average age (range)	44 (19-78) years
Average disease duration (range)	32.5 (6-72) months
Therapy	39 with 5-ASA, 2-3 g daily, 4 with SASP, 2 g daily.

5-ASA, 5-aminosalicylic acid; SASP, sulfasalazine; SCCI, Simple Clinical Colitis Activity Index.

### CLE vs. histology

None of the 9 grade A patients’ histology showed active inflammation. One of the 9 grade B patient’s GI score was 4.0. Seventeen of the 20 grade C patients’ GI scores were more than 3.0. And none of the 5 grade D patients’ GI scores was lower than 3.1. The GI scores of groups C and D were significantly higher than those of groups A and B (P < 0.001), but there was no significant difference between groups A and B (P = 0.079) or group C and D (P = 0.514). There was excellent agreement between real-time CLE and conventional histology (kappa = 0.812). The results were illustrated in Table 3.

**Table 3:** CLE vs. histology

**CLE grade**	**GI score ≤3**	**GI score >3**	**Total**
A	9	0	9
B	8	1	9
C	3	17	20
D	0	5	5
Total	20	23	43

GI scores of group C and D were significantly higher than those of group A and B (P < 0.001), but there was no significant difference between groups A and B (P = 0.079) or groups C and D (P = 0.514). There was excellent agreement between real-time CLE and conventional histology (kappa = 0.812).

GI, Geboes Index, CLE, confocal laser endomicroscopy.

### Relapse during follow-up

During the follow-up period, a total of 18 patients reported relapses with a SCCAI score of 5 or more. Those who relapsed were younger (37.4 ± 15.2 vs. 49.1 ± 18.0 [mean ± SD] years, P = 0.031) and had longer duration (41.1 ± 19.9 vs. 26.2 ± 18.9 [mean ± SD] months, P = 0.017) than those who remained quiescent (SCCAI score of <5).

**Figure 2:**
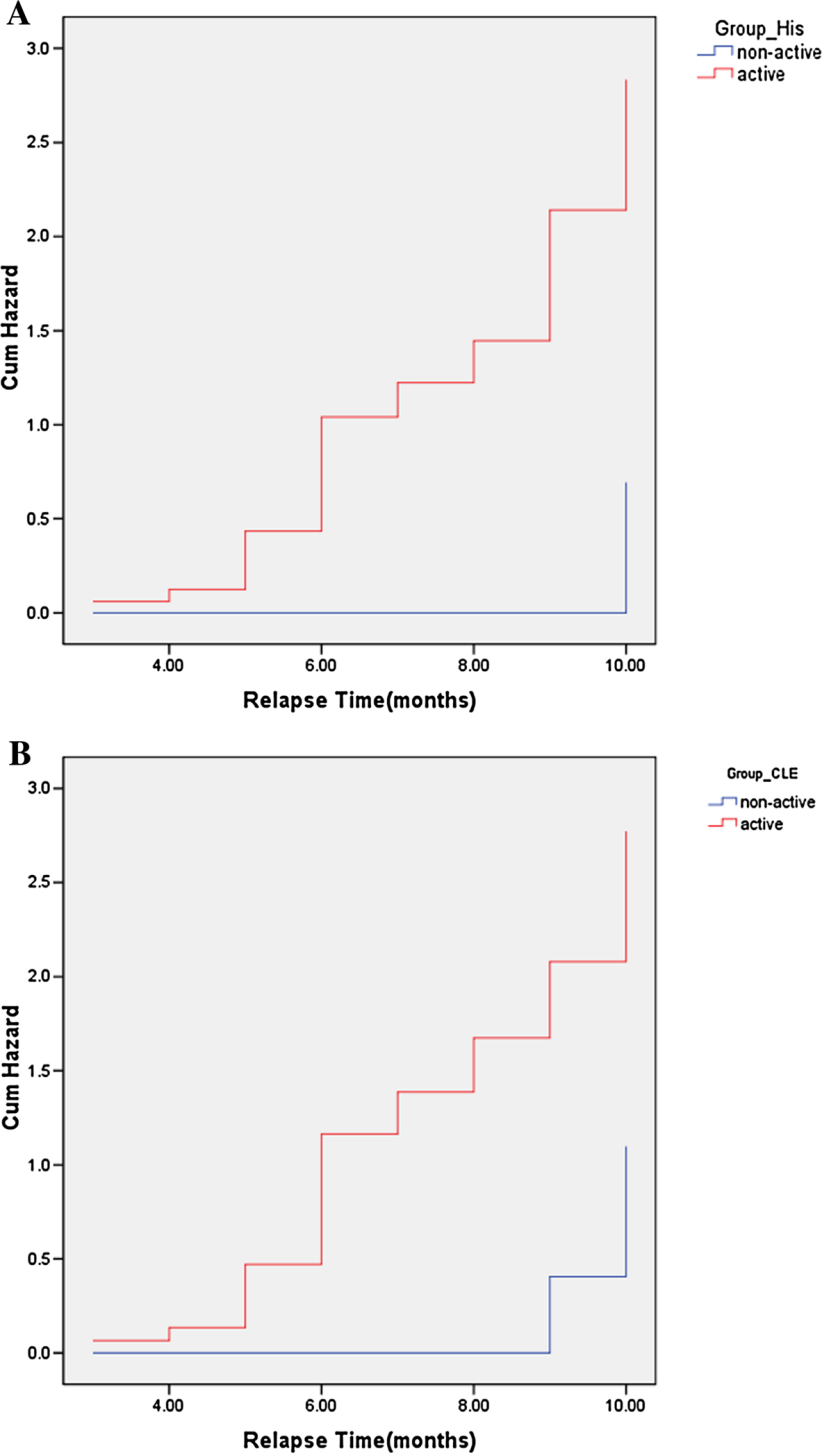
**Cumulative hazard ratio during the 12-month follow-up. A**: Cumulative hazard ratio of relapse with regard to conventional histology. **B**: Cumulative hazard of relapse with regard to real-time confocal laser endomicroscopy.

Patients who relapsed had higher GI scores (mean ± SD) than those who remained quiescent (3.82 ± 0.80 vs. 2.47 ± 0.87) at baseline. Accepting a GI score of >3.0 as the hallmark of active inflammation, the sensitivity, specificity, and accuracy of conventional histology in predicting relapse were 70.6%, 90%, and 79.1%, respectively. Patients with a GI score of >3.0 at baseline were more likely to relapse than those with a score of ≤3.0 (16/23 vs. 2/20, P < 0.001). The cumulative relapse hazard ratio during the 12-month follow-up is illustrated in Figure 2A with regard to conventional histology.

For real-time CLE classification, none of 9 grade A patients, 2 of 9 grade B patients, 11 of 20 grade C patients, and all 5 grade D patients relapsed during follow-up. Relapse rates among the four grades were significantly different (P < 0.001). Accepting grade C and D as active inflammation, the sensitivity, specificity, and accuracy of CLE in predicting relapse were 64%, 88.9%, and 74.4%, respectively. The cumulative relapse hazard ratio during the 12-month follow-up is illustrated in Figure 2B with regard to real-time CLE.

## Discussion

Treatment of inflammatory bowel disease (IBD) depends on long-term anti-inflammatory agents, such as 5-ASA, immunosuppressant and newly developed anti-tumor necrosis factor agents. The concept of mucosal healing, which indicates less relapse rate, has been incorporated into the assessment of patients with UC receiving anti-inflammatory treatment. However, there is no standard definition of mucosal healing. Neither endoscopic nor histological definition has been widely accepted [7]. Studies show that patients with histological evidence of active inflammation have a higher relapse rate despite their clinical and endoscopic remission [4,18]. Our study used CLE as the in vivo optical biopsy method to assess of inflammatory activity and predict relapse for patients with both clinical and endoscopic remission. Both in vivo optical and conventional histology identified a considerable proportion of patients with active inflammation whose clinical and endoscopic conditions were quiescent, and these patients were more likely to relapse during a 6-month follow-up.

Use of CLE in UC mainly focuses on two aspects: detection of intraepithelial neoplasia [19] and assessment of inflammatory activity. Previous reports have proved its accuracy in assessment of inflammatory activity compared with conventional histology [11-13]. Our previous study also showed that CLE is superior to conventional white-light endoscopy in assessing inflammatory activity [13]. Consequently, it is desirable to use CLE to predict relapse of UC, which may lead to more cost-effective treatment and surveillance strategies.

Kiesslich et al. were the first to use CLE to evaluate the relationship between local barrier dysfunction and relapse in IBD patients. They found that increased epithelial gaps and fluorescein leakage in the small intestine of IBD patients were associated with a higher relapse rate, with a sensitivity of 62.5%, a specificity of 91.2%, and an accuracy of 79% [20]. Increased gaps in the small intestine of IBD patients were also identified by probe-based CLE [20]. This study shows that grades C and D have a sensitivity of 64%, a specificity of 88.9%, and an accuracy of 74.4% in predicting relapse, which is comparable to the study of Kiesslich et al. [20].

What are the differences of this study from the previous ones? First, our study chose the distal colon as a target, which avoided the influence by terminal ileum intubation failure (16/135) as previously reported [20]. Second, a validated four-grade CLE classification was applied in this study. Compared with the systematic Mainz’s CLE classification which involves the assessment of crypts, cell infiltration, and vessels [11], this classification might be easier to learn for the beginners using CLE. In this study, the fluorescein leakage into the crypt lumen was incorporated into the original classification published previously [13], which indicates local barrier dysfunction.

The limitations of this study are as follows: first, although all the included patients were both clinically and endoscopically quiescent at baseline, assessment of relapse only depended on clinical assessment. Although the SSCAI is a validated scoring system, the questionnaire-based assessment from the patients’ perspective may differ from the doctor’s assessment, colonoscopy, or histological results [17]. Second, the duration of follow-up was 6 months, which is shorter than in previous trials. However, the predictive value of CLE in this study is similar to trials with a duration of 12 months [20]. Third, CLE cannot currently reliably identify different cell types, such as neutrophils or lymphocytes. Assessment of inflammatory activity can only be based on indirect parameters, such as crypt architecture and fluorescein leakage [21]. And finally, but by no means least, the sample size was small, which made multifactor analysis difficult, such as the influence of smoking, maintenance therapy drugs, and diet. Therefore, the results should be interpreted with caution.

## Conclusions

In conclusion, inflammatory activity assessment and prediction of relapse of UC using CLE are accurate compared with conventional histology.

## Competing interests

The authors declare that they have no competing interests.

## Authors’ contributions

Acquisition of funding: C-QL, YQ-L. Study design: C-QL, Y-QL. Drafting of manuscript: C-QL, JL, RJ, ZL. Interpretation of intellectual materials: C-QL, JL, RJ, ZL, X-JX. Supervision of study protocol: Y-QL. All authors read and approved the final manuscript.

## Pre-publication history

The pre-publication history for this paper can be accessed here:

http://www.biomedcentral.com/1471-230X/14/45/prepub
